# Factors Affecting Birth Weight of a Newborn – A Community Based Study in Rural Karnataka, India

**DOI:** 10.1371/journal.pone.0040040

**Published:** 2012-07-05

**Authors:** Chandra S. Metgud, Vijaya A. Naik, Maheshwar D. Mallapur

**Affiliations:** Department of Community Medicine, KLE University, Jawaharlal Nehru Medical College, Belgaum, Karnataka, India; Lund University Hospital, Sweden

## Abstract

**Background:**

Low birth weight (LBW) is a major public health problem in many developing countries, especially so in India. Although we do not know all the causes of LBW, maternal and environmental factors appear to be significant risk factors in its occurrence.

**Objectives:**

To know the factors affecting the birth weight of a newborn and to estimate the prevalence of LBW.

**Methods:**

The present study was carried out amongst 1138 pregnant women and their newborns residing in area covered by Kinaye Primary Health Centre in rural Karnataka, India. The study was conducted from 1^st^ June 2008 to 31^st^ December 2009.

**Results:**

The mean birth weight of newborns was 2.6 kg with a range of 1.2 to 3.8 kg. The prevalence of LBW was 22.9%. Among the studied risk factors, 25 of them were significantly associated with the birth weight of a newborn on univariate logistic regression analysis. Maternal education [Odds Ratio (OR) 3.2], exposure to passive smoking [OR 2.3], age at first pregnancy ≥25 years [OR 3.6], birth interval <2 years [OR 2.4], previous history of LBW baby [OR 3.3], weight gain ≤4 kg during pregnancy [OR 7.0], maternal weight at last week of gestation ≤45 kg [OR 2.3], pregnancy induced hypertension [OR 3.3], high risk pregnancy [OR 3.6] and late antenatal registration [OR 3.6] emerged as significant risk factors on multivariate analysis.

**Conclusion:**

The problem of LBW is multidimensional, and hence, we need an integrated approach incorporating medical, social, economical and educational measures to address this issue.

## Introduction

Intrauterine growth and development is one of the most vulnerable process in human lifecycle and its aberrations can result in lasting profound influence in later life. In the context of developing countries, intrauterine growth has been invariably assessed by birth weight. The birth weight of an infant is a reliable index of intrauterine growth and also a sensitive predictor of newborn’s chances of survival, growth and long term physical and psychosocial development. With the availability of fairly accurate infant weighing devices all over the world, birth weight and its determinants have come under intense global scrutiny. As a result, it is now acknowledged that many factors can influence the duration of gestation or the rate of intra-uterine growth.

Low birth weight (LBW) has been defined as a birth weight of less than 2.5 kg regardless of gestational age [1]. World Health Organisation (WHO) estimates that globally, out of 139 million live births, more than 20 million LBW babies are born each year, consisting 15.5% of all live births, nearly 95.6% of them in developing countries [2]. Infants who weigh less than 2.5 kg at birth represent about 26% of all live births in India and more than half of these are born at term. LBW infants are 40 times more likely to die within first four weeks of life than normal birth weight infants. Half of all perinatal and 1/3^rd^ of all infant deaths occur in babies with LBW [1].

Majority of studies done in India on determinants of LBW are hospital based. Data from hospitals is generally associated with a certain degree of uncertainty and bias. Those which are rural based studies most of them were done in an antenatal clinics situated at Rural Health Training Centres or record based. Hence, this study was undertaken in a rural community to know the risk factors for birth weight of a newborn. Each study has assessed only few or groups of risk factors that might influence the duration of gestation or intrauterine growth. The present study has taken into consideration all the risk factors and highlightened important independent factors of LBW through multiple regression analysis adjusting for gestational age.

## Methods

The present study was carried out amongst the pregnant women and their newborns residing in an area covered by Kinaye Primary Health Centre (PHC) in rural Karnataka, India which caters to a population of 58,980. Sample: In our study 61 risk factors were studied together and each factor had different prevalence rate. Among them history of low birth weight was one of the important risk factor [1]. The sample size was calculated based on it and using the formula n = 4 pq/d^2^, where n = sample size, p = prevalence of history of low birth weight = 26%, q = 100–p = 100–26 = 74, d = relative error, 10% of p, 10% of 26 = 2.6, therefore n = 4X26X74/2.6X2.6 = 7696/6.76 = 1138. The Ethical clearance was obtained from KLE University Ethical Committee. Data collection: Training of Health Worker (Female) [HW (F)] was undertaken for a period of 3 months and the aim was to make them get acquainted with the study protocol and was conducted in two sessions. The first session was held at the Kinaye PHC. The training components were: completion of data collection instrument, obtaining informed consent, antenatal examination, recording birth weight and investigation. The second training session was held in the respective sub-centre of the HW (F). Three antenatal women were selected from each sub-centre and the HW (F) was made to demonstrate all the elements of training in front of the researcher. When all the components of training were found satisfactory by the investigator as per the check list, the training was taken as completed. During the study period, at the end of every 3 months 10% of data collected by HW (F) was selected randomly for cross-checking the reliability, validity and accuracy.

The pregnant women were enrolled before 16 weeks of gestation. Written Informed consent was obtained from all the study participants at the time of enrollment. For illiterates the informed consent form was read to them in their vernacular language. After understanding the study and if they agreed to participate then left thumb impression was obtained. The data was collected by interview using a pre-designed and pre-tested proforma. Every enrolled woman had 3 contact examinations. During the first visit maternal socio-demographic information was collected along with obstetric history and antenatal care. Maternal height and weight, blood pressure recording and haemoglobin estimation was also done. Second visit was carried out in the third trimester (36 to 37 weeks). The data was collected regarding antenatal care, weight gain, development of high risk pregnancy, dietary history (24 hour recall method) and medical illness. Blood pressure was recorded and haemoglobin estimation was done again. The last visit was after the delivery for recording of birth weight and other relevant information. Investigator ensured that the weighing machines and the weighing procedure were standardized before the start of the study. Software SPSS 12 (trial version) was used for data analysis. Best fit model was used to access the independent effect of the factors using stepwise method.

## Results

Out of 1138 pregnant women studied, 981 (86.2%) were between 20–29 years, 78 (6.9%) ≥30 years and 79 (6.9%) were teenage pregnancies. Majority 976 (85.8%) were Hindus and most of them 769 (67.6%) belonged to classes III and IV according to Modified B G Prasad’s socio-economic status classification [3], [4]. Most of them 984 (86.5%) were literates and the majority 761 (66.9%) were housewives. The mean ± SD calorie and protein intake of the study subjects per day was 1510.7±342.3 Kcal and 47.7±11.5 gm respectively. Only one habit i.e. pan chewing with tobacco in 49 (4.3%) pregnant women was noted and 207 (18.2%) of them were exposed to passive smoking in the home during pregnancy. Body Mass Index (BMI) was normal in 702 (61.7%) pregnant women and 57 (5.0%) were underweight.

Among 1131 registered pregnant women, 740 (65.4%) had done antenatal (AN) registration in the 1^st^ trimester. Majority 922 (81.0%) women had made ≥3 AN visits. Among 1125 who received iron & folic acid (IFA) tablets, 765 (68.0%) pregnant women took ≥100 tablets. Under Reproductive and Child Health program 100 tablets of iron and folic acid (each tablet containing 100 mg elemental iron and 0.5 mg of folic acid) are given to pregnant women during the course of pregnancy. In our study, 290 (25.5%) women gained weight ≤4 kg and only 21 (1.8%) more than 10 kg. Out of 304 pregnant women who were high risk pregnancy, 259 (22.8%) had one high risk factor, 43 (3.8%) had two and 2 (0.2%) had three high risk factors. The important high risk factors noted in our study were: bad obstetric history in 76 (21.7%), previous caesarean section among 54 (15.4%), Rh. negative status in 52 (14.9%), pregnancy induced hypertension (PIH) among 40 (11.4%), etc. The mean ± SD birth weight of newborns was 2.6±0.4 kg with a range of 1.2 to 3.8 kg. The prevalence rate of LBW in our study was 263 (22.9%).

Review of various studies [2], [5] have revealed 71 modifiable risk factors for birth weight of a newborn. Out of them 10 factors could not be studied because of feasibility and 11 were absent in our study population. Among the studied risk factors, 25 were significantly associated with birth weight on univariate logistic regression analysis. They were: maternal education [crude Odds Ratio (OR) 2.4, 95% confidence interval (CI) 1.4–4.1], p 0.001], husband’s education [crude OR 3.5, (95% CI 1.6–7.8), p 0.002], exposure to passive smoking from husband [crude OR 2.0, (95% CI 1.3–3.2), p 0.002], age at first pregnancy ≥25 years [crude OR 2.1, (95% CI 1.3–34.5), p 0.001], BMI <18.5 [crude OR 2.7, (95% CI 1.5–4.9), p 0.001), maternal height ≤140 cm [crude OR 6.8, (95% CI 3.5–13.3), p 0.001), weight gain ≤4 kg during pregnancy [crude OR 6.0, (95% CI 3.7–9.6), p 0.001), maternal weight at last week of gestation ≤45 kg [crude OR 3.6, (95% CI 2.6–5.0), p 0.001), PIH [crude OR 3.9, (95% CI 2.1–7.3), p 0.001), grand multipara [crude OR 3.6, (95% CI 1.7–7.6), p 0.001), inter-pregnancy interval <2 years [crude OR 2.4, (95% CI 1.6–3.5), p 0.001), high risk pregnancy [crude OR 5.1, (95% CI 2.8–9.4), p 0.001), previous LBW baby [crude OR 4.8, (95% CI 2.0–11.6), p 0.001) and ≥2 abortions [crude OR 3.3, (95% CI 1.3–8.6), p 0.016) and institutional deliveries at tertiary health centre [crude OR 2.2, (95% CI 1.5–3.4), p 0.001).

The percentage of LBW babies was 50.0% (n = 9) in the ≥35 years age group, followed by 33.3% (n = 20) in 30–34 years age group. The least percentage of LBW was noted in the age group of 20–24 years 19.9% (n = 130). As the maternal age increased the chances of having LBW baby also increased (crude OR 4.0, 95% CI 1.6–10.3, p 0.004). A statistically significant relationship was found between the calorie intake (crude OR 4.9, 95% CI 1.7–14.1, p 0.003) and birth weight of the newborn when compared with protein intake (crude OR 2.1, 95% CI 1.2–3.7, p 0.007). Out of all the registered mothers’ outcome was better in mothers who got booked in the 1^st^ trimester. Similarly, the birth weight of babies seemed to be influenced significantly by the number of antenatal visits made by the mother (crude OR 2.9, 95% CI 2.1–4.0, p 0.001). Unregistered mothers had 11 times higher risk of having a LBW baby in comparison to those who had 3 or more visits. If the women did not consume IFA tablets during pregnancy the chances of having LBW baby was 8 times in comparison with those who consumed ≥100 tablets and it was reduced to 2 times if they consumed 50 to 100 IFA tablets. Maximum 80.0% (n = 4) number of LBW babies were born to mothers’ with haemoglobin level <7 g/L (severe anaemia) in the 3^rd^ trimester. When compared with mothers’ who had severe anaemia in the 1^st^ trimester, the risk of having an LBW increased from 5 to 20 times. These 25 risk factors identified were subjected for multivariate logistic regression analysis and 12 factors emerged as the most important risk factors for birth weight in our study area (Table 1).

**Table 1:** Univariate and Multivariate Logistic Regression Analysis.

Variables	Crude OR 95% CI	Adjusted OR 95% CI
**Maternal education**		
Illiterate	2.4 (1.4–4.1)*	3.2 (1.0–10.3)***
Primary^a^	1.5 (0.9–2.4)	2.9 (1.1–8.0)***
Secondary^b^	1.1 (0.7–1.7)	1.9 (0.7–4.9)
**Post SSLC** c **(Ref)**		
Graduate^d^	1.2 (0.4–3.6)	1.8 (0.2–22.2)
**Exposure to passive smoking**		
No (Ref)^e^		
Husband	2.0 (1.3–3.2)**	2.3 (1.1–4.9)***
In-laws & Others	1.1 (0.7–1.8)	1.2 (0.6–2.8)
**Age at 1^st^ pregnancy (in years)**		
<20	1.1 (0.8–1.5)	0.7 (0.8–1.3)
20–24 (Ref)		
≥25	2.1 (1.3–34.5)*	3.6 (1.2–10.8)***
**Birth interval** f **(in years)**		
<2	2.4 (1.6–3.5)*	2.4 (1.3–4.6)**
≥2 (Ref)		
**Previous LBW baby (number)**		
No (Ref)		
1 or ≥2	4.8 (2.0–11.6)*	3.3 (1.1–9.4)***
**Weight gain during pregnancy (in kg)**		
≤4	6.0 (3.7–9.6)*	7.0 (3.2–15.4)*
5–7	1.2 (0.7–1.9)	1.0 (0.5–2.2)
8–10 (Ref)		
>10	2.2 (0.7–6.5)	2.1 (0.4–10.2)
**Maternal weight at last week of gestation (in kg)**		
≤45	3.6 (2.6–5.0)*	2.3 (1.2–4.3)**
>45 (Ref)		
**Pregnancy induced hypertension**		
No (Ref)		
Yes	3.9 (2.1–7.3)*	3.3 (1.0–10.5)***
**High risk pregnancy** g		
No (Ref)		
1 risk factor	4.4 (3.2–6.0)*	3.6 (1.9–6.7)*
>1 risk factor	5.1 (2.8–9.4)*	3.7 (1.1–11.9)***
**Antenatal registration**		
No	9.0 (1.7–47.6)**	13.3 (0.5–33.3)
1^st^ trimester^h^ (Ref)		
2^nd^ trimester^i^	1.4 (0.7–2.8)	0.8 (0.5–1.4)
3^rd^ trimester^j^	1.0 (0.7–1.4)	3.6 (1.0–13.7)***

a Studied from first to seventh class.

b Studied from eighth to tenth class.

c Studied after tenth class or pre-university education.

d Awarded University degree in any speciality (Ref)^e^ Reference category.

f Gap between this and the previous pregnancy (excluding primiparas mothers).

g One which is complicated by factor or factors that adversely affects the pregnancy outcome.

h Conception to completion of 12 weeks of gestation.

i Over 12 weeks of gestation to completion of 28 weeks of gestation.

j Over 28 weeks of gestation. OR, Odds Ratio; CI, Confidence Interval. *p<0.001, **p<0.01, ***p<0.05.

## Discussion

The prevalence rate of low birth weight in the study was 22.9% and the mean birth weight was 2.6±0.4 kg. Majority of the studies done in rural areas of India had the same magnitude of the problem of LBWs [6]–[9]. But, one study done in Ballabgarh had the prevalence rate of LBW as low as 8.8% and another study conducted in West Bengal as high as 31.3% [10], [11]. Both these studies did not have adequate sample size. In the rural studies the mean birth weight of newborn ranged between 2.6±0.5 to 2.8±0.4 kg [6], [7], [9]–[11]. Maximum of the hospital based studies had the prevalence rate of LBW more than 30% and the mean birth weight of newborn ranged between 2.5±0.4 to 2.8±0.4 kg [12]–[14]. The prevalence rate of LBW is higher and also the mean birth weight of newborn is lesser in most of the hospital based studies. The reason may be most of the high risk pregnancies deliver in tertiary health care centres. According to National Family Health Survey –3, among the 34% of births that were weighed at birth, over one in five (22%) were of LBW. The proportion weighing <2.5 kg is slightly higher in rural areas (23%) than in urban areas (19%) [15].

Different studies have revealed that significantly associated risk factors for the birth weight of a newborn vary according to the geographical location and the study population. A prospective longitudinal study was carried out in the rural areas of district Ambala, Haryana. The bivariate analysis showed a significant association between the birth weight of baby and the maternal age, maternal education, per capita income of the family, time of antenatal registration, number of antenatal visits, physical work during pregnancy, height and weight in pregnancy. A significant association between the calorie and protein intake with the birth weight of babies was also observed [7]. The determinants of LBW are similar to our study except socio-economic status (p>0.05). A community based study was conducted in the rural areas of Dakshina Kannada district of Karnataka, Manipal. Significant determinants of LBW obtained by the stepwise multiple logistic regression were Patriarchal family custom (OR 1.3), middle (OR 1.3) and low (OR 1.1) socio-economic status, poor sanitary condition (OR 1.6), maternal age ≥35 years (OR 2.0), first birth order (OR 1.6) and poor quality antenatal care (OR 15.8) [16]. A maternity home records based retrospective case control study was conducted in the field practice area of Kasturba Medical College, Manipal. The significant determinants identified for intra uterine growth retardation after multiple logistic regression analysis were maternal age >30 years, primiparity (p 0.018), maternal height <145 cm (p 0.007), maternal weight <45 kg (p 0.001) and anaemia in pregnancy (p 0.01) [17].

The present study revealed that maternal illiteracy, exposure to passive smoking, late child bearing, shorter inter-pregnancy interval, previous LBW baby, maternal weight, weight gain during pregnancy, PIH, high risk pregnancy and late antenatal registration were the risk factors significantly associated with the birth weight of a newborn. Interventions to reduce LBW should be specific for the targeted population and directed at the quantitatively important modifiable determinants of intrauterine growth and gestational duration. So in conclusion, comprehensive approaches which institute a combination of interventions to improve the overall health of the women are needed. Such approaches are likely to be most effective in reducing the LBW problem in India.
